# Investigated the role of community based approaches for biodiversity conservation and socio-economic development in Bale Mountains National Park, Southeast Ethiopia

**DOI:** 10.1038/s41598-024-60177-5

**Published:** 2024-05-28

**Authors:** Hussein Gena Koricha, Mustafa Jemal Adem

**Affiliations:** 1 College of Agriculture and Natural Resources, Department of Biodiversity Conservation and Ecotourism, Madda Walabu University, Bale Robe, Oromia Ethiopia; 2 College of Agriculture and Natural Resources, Department of Natural Resource Management, Madda Walabu University, Bale Robe, Oromia Ethiopia

**Keywords:** Association, Park conservation, Perceived benefit, Perception, SWOT analysis, Environmental sciences, Environmental social sciences

## Abstract

Community based conservation approaches are the holistic way to ensure appropriate biodiversity conservation and livelihood improvement in many protected areas across the world. However involvement of local community in conservation activities and benefit sharing in many protected areas are not well implemented. The purpose of this study is to investigate the role of community based conservation approaches for biodiversity conservation and socio-economic development. To address the stated objectives, required data were gathered from both primary and secondary sources. The result of the study revealed that parks provide various benefits for the local communities which are found in and adjacent to the park. Regarding perceived benefits from park, there was significant difference among community based association and non-community based association at (X^2^ = 92.071, df = 1, P < 0.05) while no significant difference was observed across kebeles. In spite of variation in perception among local community on park conservation, it was revealed that the communities contribute towards conservation of the park by controlling outbreak of fire and informing wildlife attack. Regarding strengths and weakness of community based conservation approaches as the finding indicated that internal factors out weight the external factors which imply that implementing the approaches is crucial for successful conservation of the park.

## Introduction

Human beings depend on biodiversity for their survival and provision of healthy ecosystem. This is even more important for the poor people living in rural areas. For instance, 70% of the world’s poor live in rural areas depend directly on biodiversity for their livelihood. Biodiversity refers to a variety of life forms (genes, species, animals, plants and micro-organisms), ecosystems and the ecological complexes in which these components are interacting^1^. Ethiopia is a biodiversity rich country in the horn of Africa and home to 280 mammals, 861 birds, 201 reptiles, 63 Amphibians, 304 butterflies, 183 freshwater fish and around 7000 plant species^2^. Particularly Bale Eco region in which Bale Mountains National Park located is marked for immense potential of biodiversity and other natural resources^3^. It is a house for a broad-array of endemic specious of plants and animals. The Eco-region is also one of the 34 biodiversity hotspot areas in the world^3^. However, biodiversity loss has been a major concern to mankind, especially during the last quarter of the previous century^4^. During the recent times, extinction rates are ten to hundred times higher than during pre-human times^5^. As different study indicates that the underlying cause for the current decline of biodiversity are human activities such as land use change, overexploitation of species, climate change and spread of diseases which needs immediate and integrated solution like community based conservation approaches^6^.

Community based conservation initiative is a bottom-up activity promising to ensure successful conservation of biodiversity. This approaches reverses top-down and center-driven conservation by focusing on the people who bear the costs of conservation^7^. The principle of community based conservation approach in planning, implementing and evaluating programs are based on the concept that all power of decision-making rests with the people. In this context, people are involved in deciding which direction and actions to take in managing natural resources and generally considered as a people-centered participatory approach to conservation that can provide diverse environmental and socio-economic benefits^8^. Since the 1970s, the top-down exclusionary conservation model has been increasingly questioned on ethical and practical grounds^9,10^. The recognition of high administrative and social costs, concerns about long-term success of conservation, as well as an emphasis on participatory development approaches and decentralization, have brought a shift towards more decentralized and inclusive forms of natural resources management^11,12^.

Participatory approaches can be viewed as a better way to solve conflicts between local communities and protected areas, to conserve wider wildlife areas outside core protected areas and to merge conservation and development activities^13^. Ethiopia has been made a decentralization system of government reforms recently, which includes local involvement and local decision-making in resource management particularly for local people’s lives in and adjacent to protected areas. But these merely transfer state power from the center to peripheral institutions^14,15^. Contribution of community based approaches for biodiversity conservation and socio-economic development are not well determined in our country as a whole and particularly to Bale Eco region, therefore to breach these gaps this study was initiated.

## Materials and methods

### Study area descriptions

**Figure 1:**
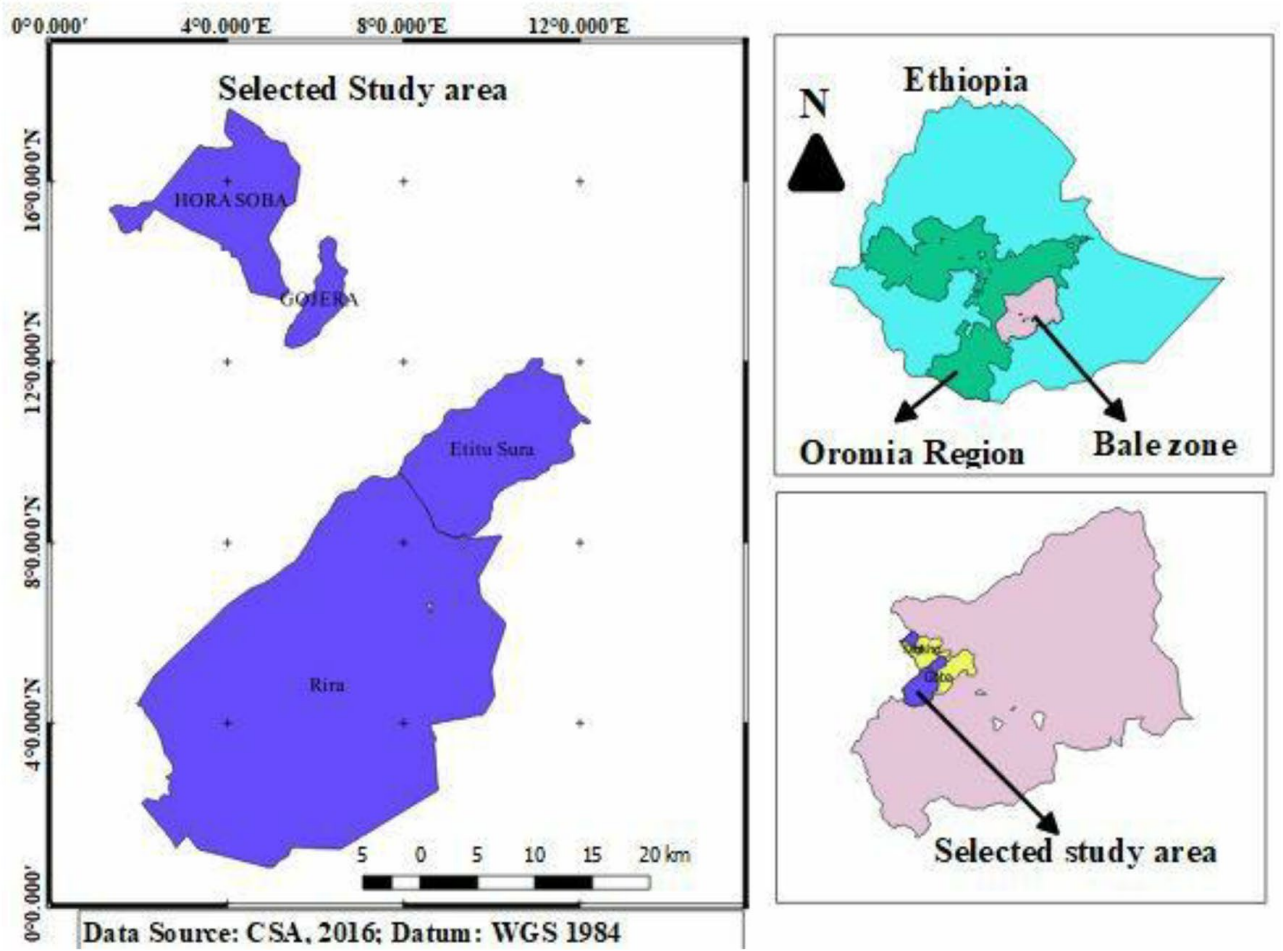
Map of the study area.

The study was conducted in Bale Mountains National Park which is located 430 km southeast of Addis Ababa in Oromia National Regional State and lies between 6° 29′–7° 10′ N and 39° 28′–39° 57′ E. The park belongs to the Bale-Arsi massif, which forms the western section of the south-eastern Ethiopian highlands covering 2150 km^2^^16,17^. It is part of Conservation International’s Eastern Afromontane Biodiversity Hotspot Area^18^, and is one of the Important Bird Areas (IBAs) designated in 1974 in Ethiopia. The Park was established by the Ethiopian Wildlife Conservation Organization (EWCO) with the primary objective of conserving the wildlife (endemic species like the Mountain Nyala (*Tragelaphus buxtoni*) and the Ethiopian wolf (*Canis simensis*) and other valuable natural resources in the area^19^. It protects a broad range of habitats from 1500 m asl in moist montane forest and ericaceous shrub land to 4370 m asl in Afro- alpine habitat on the Sanetti plateau^16^. The park is divided into five distinct and unique habitats. These are the Northern Grasslands (Gaysay Valley), Northern Woodlands (Park Headquarters), Afro-alpine Meadows (Sanetti Plateau), Erica Moorlands, and the Harenna Forest^17^. The park is also one of the remarkable ecotourism potential areas in Ethiopia (Fig. 1).

### Methods of the study

In order to achieve the objectives of the study, data were gathered from both primary and secondary sources. The primary data was collected using households survey, focus group discussions and key informant interview with different social group and institutions and direct observation. Whereas as secondary data collection methods were review of information pertaining about community based conservation approaches and Bale Mountain National Park from published materials such as books^16^, journals^17,19,21,22^, and reports^2,3^.

### Sampling techniques and size

To select sample households for the survey data, the researcher employed a multi-stage sampling procedure combining purposive and random sampling techniques. First the study woredas (Dinsho and Goba) were selected purposively since the park is mainly found in these two Woredas. Hence, the study populations are the local communities living in the two study woredas, and households in these woredas were considered as the survey population.

In the second stage of the sampling procedure, two kebeles from each woredas were purposively selected due to existence of community based associations and more of contact with the park after preliminary survey was conducted. On third stage total households of sampled kebeles were stratified into communities based and non-community based associations and sample households were selected randomly from each stratum. Hence, 187 sample sizes from total household were determined using Yamane, 1986 formula modified by Israel 2012 (Table 1).

$$n=N/1+ {\mathrm{N }({\text{e}})}^{2},$$

where n= sample size, N= total population size, e= marginal error, 1=constant number.

**Table 1:** Sample size of the households.

No	Name of kebeles	Name of Woredas in which Kebeles are found	Households size	Sample size at 7% precision level
1	Hora-Soba	Dinsho	750	59
2	Gojora	Dinsho	336	27
3	Ititu-Sura	Goba	576	46
4	Rira	Goba	700	55
Total			2362	187

Source: own survey, 2020.

Accordingly, from each kebeles proportional sample size at precious level of 7% was calculated as follow;

### Methods of data analysis

Data from the household questionnaires was coded and run to statistical package for social science (SPSS) version 22 and analyzed using descriptive statistics and responses compared using chi-square (χ^2^) tests of independence. One way ANOVA test were used for mean comparison. Moreover Strengths, weaknesses, opportunity and challenges of community based conservation association were analyzed by SWOT analysis approach considering strength and weaknesses as internal factors whereas opportunity and challenges as external factors.

## Results

Identified community based associations in the study area are community based forest management, different types of community based ecotourism association such tour guiding, horse renting, food cooking, selling hand craft products and cultural event showing which used to generate income and indirectly conserve biodiversity of the park.

### Perceived benefits from the park

The result in the Table 2, indicated that from the total respondents interviewed about 94 (98.9%) community based association members and 30 (32.6%) of non-community based association members which in total 124 (66.4%) respond that they obtain benefit from the park while about 1 (1.1%) of community based association members and 62 (67.4%) of non-community based association members which in total 63 (33.7%) of the respondents confirmed that they didn’t obtain any benefit from the park. There were significant difference among community based association and non-community based association at (X^2^ = 92.071, df = 1, P < 0.05) regarding perceived benefits from park. However result of survey revealed that benefits obtained from the park across the kebeles was not significant (X^2^ = 3.416, df = 3, P > 0.05).

**Table 2:** Perceived benefit across community based association and Kebeles.

Items	Does park benefit you? At, 95% confidence interval
Across community based association (df = 1)	X^2^ = 92.071, P = 0.001
Across kebeles (df = 3)	X^2^ = 3.416, P = 0.332

Source: own survey, 2020.

The result of survey indicated, the most dominant perceived benefits among all items was fire wood and fodder which account 80%, followed by medicinal plant (67%), bee keeping (60%) while the list perceived benefits is employment opportunities which score 13 %, (Table 3). When we look a difference of each perceived benefits across community based association, with exception of employment opportunities; all benefits were statistically significant at 95% confidence interval. This indicated members of associations were more benefited almost in all available benefit options provided in the area as compared to non-associations. This may be due to the opportunity that being the member of community based association can provide the chance of obtaining additional benefits. Focus group discussion and key informant result also indicated being a members of association make them so as to get additional benefits particularly from ecotourism activities such as tour guiding, handcraft selling, horse renting and food cooking. As they said that individual who guide tourist receive 500 Ethiopian Birr per day, cookers and horse renters obtain 500 Ethiopian Birr per day and 150 Ethiopian Birr per day respectively. Moreover, they also stated that they get opportunities to participate in different events organized by park communities such as workshop and training.

**Table 3:** Perceived benefit across CBA.

Perceived benefits	Response	Community based association	Non community based association	Positive response	Total (%)	X^2^ (df)	p-Value
Employment opportunity	Yes	9 (9.5%)	4 (4.3%)	13	7	1.898 (1)	0.168
	No	86 (90.5%)	88 (95.7%)				
Fire wood and fodder collection	Yes	63 (66.3%)	17 (18.5%)	80	42.8	43.693 (1)	0.000
	No	32 (33.7%)	75 (81.5%)				
Bee keeping	Yes	41 (43.2%)	19 (20.7%)	60		10.864 (1)	0.001
	No	54 (56.8%)	73 (79.3%)				
Medicinal plants	Yes	51 (53.7%)	16 (17.4%)	67	35.8	26.776 (1)	0.000
	No	44 (46.3%)	76 (82.6%)				
Selling hand craft products	Yes	44 (46.3%)	0 (0.0%)	44	23.5	55.721 (1)	0.000
	No	51 (53.7%)	92 (100.0%)				
Revenue sharing	Yes	22 (23.2%)	0 (0.0%)	22	11.8	24.146 (1)	0.000
	No	73 (76.8%)	92 (100.0%)				
Tour guiding	Yes	32 (33.7%)	1 (1.1%)	33	17.6	34.173 (1)	0.000
	No	63 (66.3%)	91 (98.9%)				
Horse renting	Yes	46 (48.4%)	2 (2.2%)	48	25.7	52.392 (1)	0.000
	No	49 (51.6%)	90 (97.8%)				
Food cooking	Yes	18 (18.9%)	0 (0.0%)	18	9.6	19.288 (1)	0.000
	No	77 (81.1%)	92 (100.0%)				

Source: own survey, 2020.

### Contribution of local community towards park conservations

The sampled response indicated that majority (87.2%) of the respondents were agreed that they play great role in conservation of the park’s biodiversity whereas few (12.8%) of them didn’t contribute towards park conservation and their contributions were significantly different across community based association (X^2^ = 7.334, df = 1 and P < 05) (Table 4).

**Table 4:** Roles in conservation of the park across community based association.

No	Item	Community based association	Non community based association	Frequency	Percent	X^2^ (df)	P-value
1	Contribute to the park conservation in any means	89	74	163	87.2	7.334 (1)	0.007
2	Not contribute to the park conservation	6	18	24	12.8		
Total		95	92	187	100		

Source: own survey, 2020.

**Figure 2:**
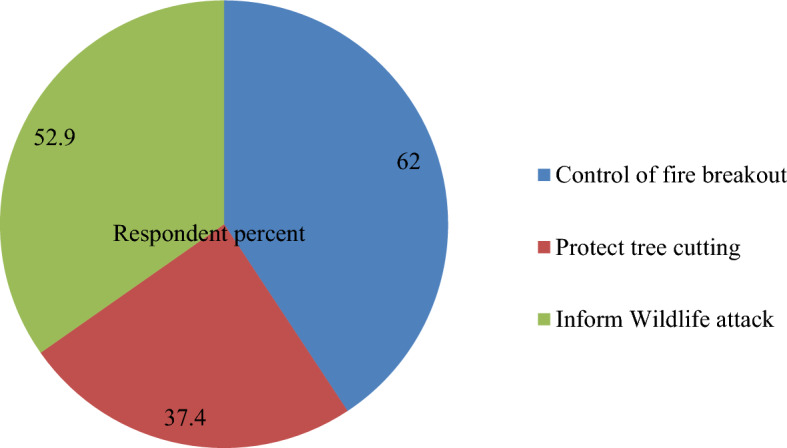
Communities roles in park biodiversity conservation.

The result in Fig. 2 indicated that majority (62%) of the respondents contribute towards conservation of the park by controlling outbreak of fire, followed by informing wildlife attack (52.9%). Besides, result from focus group discussion and key informant interviews also supports this finding. They said that know day, even if the relationship between park communities and local peoples were not as such strong, from the beginning they consider the park as their own resource and conserve it. Additionally for a long period of time, before the area considered as park, they protect and conserve as part of their life using own traditional sanction.

### Perceptions of local community towards park conservation

The result in Table 5 indicates that there is no significant variation across association regarding perception of considering park as the government resource than their own asset at (X^2^ = 9.831, df = 4, P > 0.05), similarly no significant variation across association about how the community can better manage the park if full responsibility is given to them (X^2^ = 10.114, df = 4, P > 0.05). Hence, about 57.9% of community based association members and about 44.6% of non-community based association members perceive and consider park as their own resource not as the government resource. Despite the similarity in perception there is a percentage difference across two associations. However significant variation was observed across association in terms of relations between park and community is cordial (X^2^ = 44.862, df = 4, P < 0.05) and also feeling about the way this park is being managed (X^2^ = 68.374, df = 4, P < 0.05). This implies members of association has positive outlook regarding the relationship between park communities and way of park being managed. Result from focus group discussion and key informant interviews also indicated that they have mixed response in terms of perception towards the park conservation activities. It may be depend on perceived benefits and conflicts among communities. They said that the relationship between park community and local peoples was not good. The main reason they stated are when park animal damage our crop they do not compensate balanced payment and also not actively participate in management activities of the park, except few individuals.

**Table 5:** Local people’s perception across community based association.

Variables	Response at community based association in percentages	Response at non community based association in Percentages	X^2^ (df)	P-value
Peoples consider the park as liability than asset	Mean = 3.48, SD = 0.944; n = 95	Mean = 3.02; SD = 1.089, n = 92	9.831 (4)	**0.055**
Strongly agree	3.2	8.7		
Agree	15.8	29.3		
Neither agree nor disagree	16.8	15.2		
Disagree	57.9	44.6		
Strongly disagree	6.3	2.2		
Community can better manage the park if given full responsibility	Mean = 1.93, SD = 0.854; n = 95	Mean = 2.25, SD = 1.125; n = 92	10.114 (4)	**0.06**
Strongly agree	28.4	26.1		
Agree	60.0	47.8		
Neither agree nor disagree	4.2	3.3		
Disagree	5.3	20.7		
Strongly disagree	2.1	2.2		
Relations between park and community is cordial	Mean = 2.42, SD = 1.116; n = 95	Mean = 3.40, SD = 0.915; n = 92	44.862 (4)	**0.001**
Strongly agree	18.9	2.2		
Agree	45.3	18.5		
Neither agree nor disagree	15.8	20.7		
Disagree	14.7	54.3		
Strongly disagree	5.3	4.3		
How do you feel about the way this Park is being managed	Mean = 1.87, SD = 0.802; n = 95	Mean = 3.21, SD = 1.033; n = 92	68.374 (4)	**0.000**
Very happy	31.6	5.4		
Happy	56.8	25.0		
Neither happy nor unhappy	4.2	16.3		
Unhappy	7.4	50.0		
Very unhappy	0.0	3.3		

Source: own survey, 2020. Significant values are in bold.

### SWOT analysis of community based associations

In this study SWOT analysis was conducted to assess suitability of community based conservation association. In this process, the internal factor (weakness and strengthens) and external factor (opportunities and threats) were identified and listed. For evaluation of internal factor estimate matrix (IFEM) and external factor estimate matrix (EFEM) were used. In formulating the matrices, each factor was evaluated by giving a weight between zero (non- important) to1 (most important) such a way that the total point in each matrix was calculated. Further, each factor was scored with a number between one and four (1 = Major weakness; 2 = Minor weakness; 3 = Minor strength; 4 = Major strength, for internal matrices) whereas 1 = Major threats; 2 = Minor threats; 3 = Minor opportunities; 4 = Major opportunities, for external matrices.

After the weighted and score have been determined, the weighed score has been given to each factor which is useful in assessing the priorities/importance of each factor. In IFEM the total of weighted score (importance) with the value of more than 2.5 indicates strength are more than weakness. Similarly, in the case of EFEM the totals weighted score with the value of more than 2.5 means opportunities are more than threats. Weighted score for the strengths and weakness IFEM and opportunities and threats are tabulated in Tables 6 and 7.

**Table 6:** Weighted score for the strengths and weakness.

No		Weight	Score	Weighted score
Strengths				
1	Control illegal activities	0.259	4	1.036
2	Advise and punishment of members	0.195	3	0.585
3	Awareness about conservation	0.225	4	0.9
Sub total		0.679		2.52
Weakness				
1	Poor coordination among members of association	0.110	2	0.22
2	Members turnover and low regular meeting	0.083	2	0.166
3	Less response to the problems occurred in association	0.046	1	0.046
4	Limited income sources	0.085	2	0.17
Sub-total		0.323		0.602
Total		1.000		

Source: own survey, 2020.

**Table 7:** Weighted score for opportunities and Threats.

No		Weight	Score	Weighted score
Opportunities				
1	Presence of community based conservation initiatives	0.148	4	0.592
2	The prevalence of alternative job opportunities	0.15	4	0.6
3	Presence of diverse natural resources in the area	0.195	4	0.78
4	The presence of different NGOs, those work on conservation of the park	0.13	3	0.39
5	Presence of community relationships	0.117	3	0.35
Sub-total		0.74		2.67
Threats				
1	Occurrence of anthropogenic factors that cause the loss of biodiversity of the park	0.086	2	0.172
2	Political instability and security problems	0.049	1	0.049
3	Low recognition and commitment from concerned body	0.079	2	0.158
4	Poor promotion of the area and association	0.054	1	0.054
Sub-total		0.26		0.433
Total		1.000		

Source: Own survey, 2020.

Thus, by comparing internal and external factors in the matrix of strengths, weaknesses, opportunities, and threats (SWOT) acceptable strategies were formulated which are as follows; Internal factor estimate matrices regarding strengthens three factors were identified (Table 7). The weight allocated for these factors where between 0.195 and 0.259 and score ranged between 3 and 4. When considering weakness four factors were detected with the lowest weight 0.046 and the highest 0.110 with score range between 1 and 2. The final weighed score for strength was 2.521 while that of weakness was 0.602. This implies that strength were more than weakness. External factor estimate matrices; there were five factor pertaining to opportunities with weight between 0.117 and 0.195 and score between 3 and 4, whereas four threats were determined with lowest weight of 0.049 and highest weight of 0.086 with scores between 1 and 2. The final weighted score for opportunity was 2.67 while that of threats was 0.433. This indicates that opportunities more than threats (Tables 8 and 9).

**Table 8:** Strength–weakness strategies.

Strength–weakness (strategies that reduce/avoid weakness by maximizing strength)
Enhance advising and regulating mechanism for creating good coordination and commitment among members of association
Exploiting alertness of community about conservation initiatives to ensure timely response to the problems occurred in association
Supplement incentives and enhance amount of payment of services provide for community members
Empower members of the community to control illegal activities in the park to minimize the problems occurred
Aware and advice community about the importance of working together on conservation activities to reduce member turnovers.

Source: own survey, 2020.

**Table 9:** Opportunities–threats strategies.

Opportunities–threats (strategies that minimize threats by maximizing opportunity)
Widely implement and incorporate community based association in conservation activities to minimize anthropogenic factor that cause the loss of park biodiversity
Built strong relationships and create collaboration among community and local security agents to minimize the problems of political instability and security to maintain the safety of working environment
Enhance the role of non-governmental organization working on conservation area for strengthen and maximizing promotion of existing potential resource and conservation initiative
Excel and enforce implementation of existing community based conservation initiatives to enhance recognition and commitment from concerned body

Source: own survey, 2020.

## Discussion

Community based conservation approach is a win-win situation in which both conservation and community development are simultaneously achieved^20^. Moreover it plays a great role in bringing effective and sustainable biodiversity conservation in protected areas as well as improving the livelihoods of local communities. To realize these importance’s active community participations in conservation and clear benefits sharing schemes are required. Our finding revealed that more than 94% of community members were benefited from conservation of the park relatively to that of non-community based association. These results agreed with other similar study conducted by Ref.^21^ which indicated that many protected areas particularly park has a potential to contribute on the local community livelihood benefit both directly and indirectly. Similar finding was noted by Ref.^22^ that local communities have benefited a lot from the park, even if perceived benefits were vary across village depending on their proximity to the park and duration of settlements as well as level of participation in conservation activities. Report of Ref.^20^ also indicated that majority of the respondents were received benefits from protected area. Moreover another similar study conducted by Ref.^23^ also reflected that communities based conservation initiatives for park associated local peoples have the potential to significantly enhance local development and socio-economic benefits through job creation.

Community participation is the key strategy to current biodiversity conservation and management^24^. If wildlife and protected areas are to survive, it is imperative that conservation activities and communities are in harmony so that it does not constraint community livelihoods. To realize effective conservation of natural resources of the park there is need for integrative management that considers local communities stake in conservation. According to the respondent’s response majority of local peoples are participated in conservation of the park. In support of our findings, similar study conducted by Ref.^25^ indicated that, the participation of local community in natural resources management is the integration of local people to mobilize themselves to make decisions, manage their resources and control the activities that affect their lives. Moreover they argue that the local people have been over looked completely in the local community which are supposed to be involved in resource management through the process of gradually handling of harvesting and management activities of their natural resources. Furthermore other study conducted by Ref.^24^ also confirm that, even limited levels of awareness existing, local community are proactive in conservation of natural resources by controlling illegal acting towards wildlife.

Inequitable sharing benefits and costs of the conservations is an obvious challenge that needs to be appropriately addressed in the management of protected areas, as it often affects the attitudes of people towards conservation. Attitudinal studies are increasingly being used to evaluate local people’s perceptions towards conservation and enable protected area management to create appropriate strategies^26^. Similarly^27^ argue that attitude change is often the only tool available to conservationists when other approaches such as regulations are ineffective. Nonetheless, some studies show that positive attitudes alone may not directly translate into friendly conservation behavior. According to the respondent’s response there is mixed response in terms of perception towards the park conservation activities which may be depend on perceived benefits and costs they incurred. In addition to these, the suit of socio-economic variables influences the attitudes of local communities towards conservation areas. Finding by Ref.^28^ reflected that there had been many complaints by local people about the continuing problems related to their restriction of their resource use activity within the area. Similarly study conducted by Ref.^29,30^ indicated that the relationship between park and peoples were negative due to crop and livestock damage by wildlife, and restrictions imposed by the reserve authorities in collecting forest products. Moreover the pressure and conflict from conservation authorities, grazing fines, and benefits are for government. Since traditional management strategies which using fence and fine principle fail to bring successful conservation of the protected areas, the necessity of community based conservation approaches have no doubt to balance the trade- off between conservation objectives and socio-economic needs of local community living in proximity of protected areas^31^. Our finding regarding analysis of suitability of community based association indicated that organizing local community in the form of benefit sharing schemes and participating them in conservation activities ensure sustainable management of the park and also empowering communities as a resources their own assets. Moreover strength and opportunities out weights the weakness and threats, implementation of the associations are appropriate strategies for successful conservation of park’s biodiversity. Similarly^32^, reported that even though there are certain gaps in implementation of the projects, community based forest management has important role in improving the livelihoods of local communities and for sustainability forest management, if the existed weakness and threats are addressed. Moreover the finding of Ref.^33^ in their analysis of suitability of community based conservation projects indicated that there is more of success than failure regarding capacity building in local communities and ecological conservations of the area.

## Conclusion

One methods of counteracting the decline and loss of biodiversity overtime is application of community based a conservation approach which is people-centered participatory approach to conservation that can provide diverse environmental and socio-economic benefits. The study revealed that there was a variation in terms of perceived benefit between members of community based association and non- community based association. Peoples in the area contribute towards park biodiversity conservation through control of fire and informing attack of wild animals. The overall perception of local communities towards the park is both positive and negative outlook. This may depends on perceived benefits and conflicts. The SWOT analysis result indicates that internal factor estimate final weighted score for strength was 2.52 while that of weakness was 0.602 whereas external factor estimate final weighted score for opportunity was 2.67 while that of threats was 0.433. Since strength outweighs weakness and opportunity outweighs threats, implementation of community based conservation association is appropriate to ensure successful conservation of the park.

### Government’s policy support

One of the priority areas of national action towards the effective conservation, rational development and sustainable utilization of natural recourse is a national commitment through an appropriate government policy (Ethiopian Biodiversity Institute, 2008). To this end, the national policy on Biodiversity Conservation and Development is formulated based on the rationale that the conservation of biodiversity is one of the conditions of the overall socio economic development and sustainable environmental management goals. Regarding these, Article 6 of the Convention on Biological Diversity (CBD Secretariat, 2003) requires parties to develop national strategies, plans or programmes for conservation and sustainable use, and to integrate these into other relevant sectoral plans. This requirement is partially met by the current Biodiversity Strategy and Action Plan for Ethiopia. There are policies and strategies in place that address biodiversity conservation directly such as National Biodiversity Conservation and Research Policy (Approved in 1998) and the Conservation Strategy of Ethiopia (CSE) (1997) and Regional Conservation Strategies (RCSs) specific to the regions (Ethiopian Biodiversity Institute, 2008).

### Ethics declaration and consent to participate 

The study was approved by the Institutional Ethics Review Committee of Madda Walabu University, College of Agricultural and Natural Resources with Ref N0 RMU-14/80/2020. Respondents were informed about purpose of the study and oral informed consent was obtained from the study participants and their legal guardians to maintain their confidentiality which is approved by Ethics Committee of Madda Walabu University, College of Agriculture and Natural Resources with Ref. N0. RMU-14/80/2020. Respondents were informed of their right to withdraw from the study at any time with no subsequent harm for refusal of participation. All methods were carried out per relevant guidelines and regulation or declaration of Helsinki.

## Data Availability

All the data supporting the funding is contained within the manuscript, when there is in need the data set used for the study can be accessible from the corresponding author upon reasonable request.
